# 艾曲泊帕、重组人血小板生成素联合标准免疫抑制治疗重型再生障碍性贫血16例疗效及安全性分析

**DOI:** 10.3760/cma.j.issn.0253-2727.2021.12.010

**Published:** 2021-12

**Authors:** 芳芳 袁, 青兰 张, 丽娜 张, 媛媛 熊, 梦娟 李, 虎 周, 旭东 魏, 新建 刘, 永平 宋

**Affiliations:** 郑州大学附属肿瘤医院，河南省肿瘤医院，河南省血液病研究所 450008 Department of Hematology, The Affiliated Cancer Hospital of Zhengzhou University（Henan Cancer Hospital）, Zhengzhou 450008, China

**Keywords:** 贫血，再生障碍性, 艾曲泊帕, 重组人促血小板生成素, 免疫抑制治疗, Anemia, aplastic, Eltrombopag, Recombinant human thrombopoietin, Immunosuppressive therapy

## Abstract

**目的:**

评价艾曲泊帕、重组人血小板生成素（Recombinant human thrombopoietin, rhTPO）联合标准免疫抑制治疗（Immunosuppressive therapy, IST）对重型再生障碍性贫血（Severe aplastic anemia, SAA）的疗效及安全性。

**方法:**

回顾性分析2017年1月1日至2020年6月30日河南省肿瘤医院16例接受IST联合艾曲泊帕、rhTPO治疗的SAA患者，评价其疗效及安全性。

**结果:**

艾曲泊帕、rhTPO联合标准IST治疗后3个月血液学反应率为81.3％，完全血液学缓解率为37.5％，6个月血液学反应率为87.5％，完全血液学缓解率为50.0％，脱离血小板输注中位时间为35（16～78）d，脱离红细胞输注中位时间为47.5（15～105）d，血小板输注中位量为5.5（3～20）U，红细胞输注中位量为6.5（2～16）U。

**结论:**

艾曲泊帕联合rhTPO可提高SAA的IST血液学反应率，改善血液学缓解质量，且不良反应轻微。

## Abstract (en)

**Objective:**

To evaluate the efficacy and safety of combination therapy of eltrombopag, recombinant human thrombopoietin (rhTPO), and standard immunosuppressive therapy (IST) for severe aplastic anemia (SAA).

**Methods:**

A total of 16 cases with SAA treated with IST combined with eltrombopag and rhTPO were retrospectively analyzed.

**Results:**

At 3 months, the total response rate was 81.3％, and the complete hematological response rate was 37.5％. At 6 months, the total response rate was 87.5％, and the complete hematological response rate was 50.0％. The median time of platelet transfusion independence was 35 (16–78) days, the median time of red blood cell transfusion independence was 47.5 (15–105) days, the median platelet transfusion was 5.5 (3–20) U, and the median red blood cell transfusion was 6.5 (2–16) U.

**Conclusion:**

The combination of eltrombopag and rhTPO can improve the hematological response rate of IST for SAA and the quality of hematological remission with minimal toxic effects.

获得性再生障碍性贫血（Aplastic anemia, AA）是由免疫介导的骨髓破坏导致的一种骨髓衰竭性疾病，以骨髓发育不全和造血干细胞缺乏为特征。杨崇礼等[1]于1991年发布的流调数据显示我国AA年发病率为7.4/100万。AA的治疗选择取决于疾病的严重程度、患者年龄、供者的可及性以及获得治疗的机会。年轻重型再生障碍性贫血（Severe aplastic anemia, SAA）患者接受HLA同胞全相合异基因造血干细胞移植总生存（overall survival, OS）率达80％[2]–[3]。抗胸腺细胞球蛋白（antithymocyte globulin, ATG）联合环孢素A（cyclosporine A, CsA）的标准免疫抑制治疗（immunosuppressive therapy, IST）的总体反应率为30％～70％[4]–[5]。有研究表明加入重组人血小板生成素（recombinant human thrombopoietin, rhTPO）或艾曲泊帕均可提高IST疗效[6]–[7]，但仍存在完全缓解率低、复发率高等问题。而rhTPO及艾曲泊帕与血小板生成素受体（c-MPL）结合位点不同，从而不存在竞争性结合，因此艾曲泊帕与rhTPO在接受IST治疗的SAA患者中可能产生协同作用。本研究中我们将IST与艾曲泊帕、rhTPO结合使用治疗初治SAA患者，以观察艾曲泊帕与rhTPO的联合使用能否进一步提高IST疗效。

## 病例与方法

1. 病例资料：本研究回顾性分析了2017年1月1日至2020年6月30日在我院接受艾曲泊帕、rhTPO联合标准IST的16例初诊SAA患者，男8例，女8例，中位年龄30.5（11～62）岁，诊断参照《再生障碍性贫血诊断与治疗中国专家共识（2017年版）》[8]。依据Camitta标准[9]将AA按严重程度分为SAA及极重型SAA（VSAA）。16例患者均行骨髓穿刺、骨髓活检检查，并除外先天性及其他获得性骨髓造血衰竭性疾病。16例患者无HLA相合同胞供者或因年龄、合并症、患者意愿等原因不适于一线接受异基因造血干细胞移植治疗。本研究经我院伦理委员会备案批准（批件号：2016120），所有患者或其监护人已知情同意并签署知情同意书。

2. 治疗：（1）IST方案：兔抗人胸腺细胞球蛋白（r-ATG，法国赛诺菲公司）3.00～3.75 mg·kg^−1^·d^−1^或猪抗人淋巴细胞球蛋白（p-ALG，武汉生物制品所）20～30 mg·kg^−1^·d^−1^，静脉输注，连用5 d；同时给予糖皮质激素预防即刻不良反应和血清病反应，2周减停。CsA口服剂量为3～5 mg·kg^−1^·d^−1^，与ATG/ALG同时应用，1周后开始监测血药浓度，调整谷浓度为200～400 µg/L。口服CsA至少6个月，达到最佳血液学反应且至少稳定半年后开始缓慢减量。CsA治疗2～3年。

（2）rhTPO与艾曲波帕：rhTPO（沈阳三生制药公司）于ATG结束输注后开始给予，成人15 000 U，儿童7 500 U，皮下注射，每日1次，总计注射28 d或血小板恢复至50×10^9^/L时停用。艾曲泊帕于ATG/ALG输注结束后开始口服，起始用量为25 mg/d，每周增加25 mg，儿童加量至50 mg/d维持，成人加量至75 mg/d维持，血小板恢复正常后至少3个月每周递减（第1周口服6天停1天，第2周口服5天停2天，依此类推），血小板未恢复正常者继续口服直至采取其他治疗策略。

（3）支持治疗：在血常规ANC<0.5×10^9^/L时，采用G-CSF 300 µg/d皮下注射，直至ANC介于（0.5～1.0）×10^9^/L。HGB<60 g/L、PLT<10×10^9^/L时给予辐照红细胞、血小板输注支持治疗。给予伏立康唑或泊沙康唑口服预防真菌感染。

3. 疗效评价：IST后定期复查外周血常规、骨髓细胞形态学及组织活检。疗效判定参照Camitta标准[9]。完全治疗反应（complete response，CR）：脱离输血和血小板输注，HGB≥100 g/L、ANC≥1.5×10^9^/L且PLT≥100×10^9^/L；良好部分治疗反应（good partial response, GPR）：脱离输血和血小板输注，未达CR但①HGB≥80 g/L、②ANC>1.0×10^9^/L、③PLT>50×10^9^/L至少符合两条；部分治疗反应（partial response, PR）：脱离输血和血小板输注，ANC>0.5×10^9^/L，实验室检查不再符合SAA标准而未达GPR标准；无治疗反应（not response，NR）：未达PR标准。定义CR+GPR+PR为血液学反应，CR+GPR为良好血液学反应。随访：采用住院、门诊复查及电话进行随访。随访截止日期为2021年1月31日。

## 结果

1. 患者基本特征：初治SAA患者16例，男8例，女8例，中位年龄为30.5（11～62）岁，SAA 12例，VSAA 4例。HDR15基因阳性11例（68.8％），阴性5例（31.2％）。AA诊断至开始IST中位间隔时间为31（7～842）d。IST方案中，14例患者免疫抑制剂类型为ATG，2例为ALG。全部患者中位HGB为63（36～74）g/L，中位PLT为11.5（2～25）×10^9^/L，中位网织红细胞绝对计数（Absolute reticulocyte count，Ret）为23（2～53）×10^9^/L，中位ANC为0.585（0.030～0.820）×10^9^/L。

2. 近期疗效：16例患者无1例早期死亡，均能评价近期疗效。IST治疗后3个月，CR 6例、GPR 3例、PR 4例、NR 3例，血液学反应率为81.3％（其中良好血液学反应率为56.3％）；IST治疗后6个月，CR 8例、GPR 4例、PR 2例、NR 2例，血液学反应率为87.5％，其中良好血液学反应率为75.0％。

3. 造血恢复情况：共有14例患者脱离输血，脱离血小板输注中位时间为35（16～78）d，脱离红细胞输注中位时间为47.5（15～105）d，血小板输注中位量为5.5（3～20）U，红细胞输注中位量为6.5（2～16）U。

4. 不良反应：16例患者除注射局部轻微疼痛外无其他明显不良反应，无血栓和栓塞事件发生。共10例患者出现肝功能异常，应用保肝药物后好转；12例患者合并发热，经抗感染治疗后体温控制，临床评价这些不良反应与应用ATG、CsA更为相关。8例患者出现皮肤颜色变深，以颜面部为著，考虑为艾曲泊帕相关不良反应。IST后3个月和6个月骨髓组织活检均未发现网状纤维和胶原纤维增生。

5. 艾曲泊帕应用情况：16例患者服用艾曲泊帕的中位时间为7.3（3～12）个月，至末次随访共8例患者停药，其中2例停药后出现血小板轻度下降，再次服用艾曲泊帕后血小板逐渐恢复。

## 讨论

目前SAA标准的治疗方案包括异基因造血干细胞移植和IST，进行同胞异基因造血干细胞移植的SAA患者长生存率高达80％[10]–[11]。IST可使2/3的患者产生治疗反应，但复发率高，许多患者持续存在血细胞减少。使用替代的免疫抑制剂或添加造血细胞因子如G-CSF和EPO并未改善IST疗效[12]–[13]，主要原因在于EPO及G-CSF仅定向作用于髓系或红系祖细胞，而SAA患者存在造血干细胞缺陷，定向造血祖细胞缺乏。

TPO是促进血小板产生的关键调节因子，主要由肝脏合成，但也可以由骨髓基质细胞和艾曲泊帕合成，它通过结合巨核细胞上的受体c-MPL而促使血小板成熟和释放。TPO在调节巨核细胞生成和血小板产生以外的造血作用中具有多效性作用，刺激c-MPL信号通路可以克服AA患者造血干祖细胞的消耗[14]–[16]。rhTPO是利用基因工程技术，通过中国仓鼠卵巢细胞表达，经提纯后制成的全长糖基化修饰的糖蛋白分子，分子结构与天然TPO的结构完全一致，与内源性TPO具有相似的药理作用。张莉等[7]及Wang等[17]表明rhTPO可以改善接受IST的SAA患者的血液学反应并加速血小板恢复。

艾曲泊帕是一种口服血小板生成素模拟物，可与造血干/祖细胞膜受体结合促进其增殖分化，扩增体内残存造血干细胞，同时具有免疫调节、诱导免疫耐受及螯合去铁作用[18]。Townsley等[6]在一项前瞻性研究中纳入了92例初治SAA患者，采用IST联合艾曲泊帕治疗，结果表明加入艾曲泊帕后患者血液学应答率及总体缓解率均高于历史数据，患者骨髓细胞数量、CD34^+^细胞数量和早期造血祖细胞的数量均显著增加，中位随访2年，生存率为97％。杨文睿等[19]评估了艾曲泊帕联合IST在中国AA患者中的疗效及安全性，19例初治SAA患者应用艾曲泊帕，IST后3个月血液学反应率为73.6％（14/19）、完全缓解率为36.8％。艾曲泊帕结合在c-MPL配体结合口袋外部的跨膜区域，从而不与TPO竞争c-MPL的结合，并通过JAK-STAT和MAPK途径激活信号传导，在TPO水平升高的情况下仍能通过非竞争性激活c-MPL刺激造血功能，以不同于TPO的方式激活信号传导，可能与TPO发挥协同作用[20]。基于这一研究基础，我们采用艾曲泊帕+rhTPO联合IST治疗SAA患者以观察其近期疗效。本研究中共16例患者接受了艾曲泊帕+rhTPO联合IST治疗，3个月及6个月血液学反应率分别为81.3％、87.5％，良好血液学反应率分别为56.3％、75.0％，完全缓解率分别为37.5％、50.0％，明显高于我院既往接受IST联合单一rhTPO治疗的SAA患者，且高于目前文献发表的IST联合rhTPO或艾曲泊帕治疗初治SAA的近期疗效[7],[19]。

有关艾曲泊帕在SAA患者IST治疗的应用疗程目前各项研究尚无一致结论，Townsley等[6]的研究中设计了3个队列，艾曲泊帕应用的时间分别为3个月或6个月，在停用艾曲泊帕后部分患者仍继续保持稳定的计数，因此当产生一定数量的造血干细胞时，功能性造血可将血细胞计数维持在接近正常范围，而无需持续暴露于艾曲泊帕。本研究中16例患者服用艾曲泊帕的中位时间为7.3（3～12）月，6个月时获得CR的8例患者均已停药，其中2例停药后出现血小板轻度下降，再次服用艾曲泊帕后血小板逐渐恢复。至随访结束无一例患者出现骨髓造血组织纤维增生、血栓事件及克隆性血液学异常演变，但本研究样本量相对较小，尚需前瞻性的对照试验进一步验证艾曲泊帕+rhTPO联合IST治疗SAA/VSAA的疗效和安全性。
